# Duration Analysis for Coronary Artery Disease Patients With Chronic Chest Pain: An Output From Saudi Arabia

**DOI:** 10.15171/jcvtr.2015.02

**Published:** 2015-03-29

**Authors:** Mehwish Hussain, Nazeer Khan, Mudassir Uddin, Mansour M. Al-Nozha

**Affiliations:** ^1^ Department of Statistics, University of Karachi, Karachi, Pakistan; ^2^ Department of Research, Dow University of Health Sciences, Karachi, Pakistan; ^3^ Department of Research, Jinnah Sindh Medical University, Karachi, Pakistan; ^4^ Faculty of Medicine, Taibah University, Madinah Munawwarah, Saudi Arabia

**Keywords:** Chronic Chest Pain, Coronary Artery Disease, Duration Analysis, Diagnosis, Predictors

## Abstract

***Introduction:*** Coronary artery disease (CAD) is a persistent public health problem worldwide. Chest pain is one of the perceptible symptoms of the same disease. Literature has found acute chest pain as plausible risk factors for CAD. Nevertheless, none of the study has estimated duration from chronic chest pain to the diagnosis of CAD. The objective of the study was to estimate duration from chronic chest pain to CAD and to assess impact of risk factors on same duration.

***Methods:*** Data were obtained from community based study on 17,232 Saudi adults. History of patients about onset of chest pain and other risk factors were inquired. Descriptive measures were obtained by Kaplan-Meier curve. Effect of demographic and clinical factors was assessed by Cox regression models.

***Results:*** Out of 24% patients with chest pain, 21% diagnosed with CAD. The average duration was 5 years. About 12% of patients with chest pain diagnosed with CAD after one year. Advancing age, female gender, no exercise and reduced high density lipoprotein (HDL) were significantly hazardous predictors throughout duration from chest pain to diagnosis of CAD.

***Conclusion:*** The duration from chest pain to CAD was 5 years. Age, gender, exercise and HDL can be variables of concern to deteriorate hazards of CAD for patients with chest pain.

## Introduction

Chest pain is one of the perceptible symptoms of coronary artery disease (CAD).^1,2^ A systematic review of 31 countries reported the average weighted prevalence of angina in male was 5.7% and in female, it was 6.7%.^3^ The prevalence of CAD in patients with chest pain was reported to be 19.4% in urban population of Iran.^4^ Though, in primary care patients, such prevalence is estimated to be 1-2%.^5,6^ The time it takes for a patient having cardiac chest pain to the diagnosis of CAD is getting significance.^2^ Physicians are involved in predicting the diagnosis of CAD in patients with chest pain.^1,7,8^ So far, the prediction of CAD in patients with chest pain had been done on primary care patients^9,10^ or via systematic reviews^1,11^ or among patients with acute chest pain.^1,8,12^ The duration from the first onset of chest pain to CAD diagnosis has not been determined yet. Thus, this study filled the gap while estimating duration of diagnosis of CAD for those patients who encountered chest pain in past. Such analysis will help cardiologists to treat patients having chest pain with framed duration. Furthermore, risk assessment analyses assist physicians to focus more on the specified significant risk factors to avoid hazards of encountering CAD. Therefore, another objective of the study was derived to identify hazardous predictors which jeopardize the risk of shortening this time duration.

## Materials and methods

### The Data

The data were obtained from 5-years National Epidemiological Health Survey on CAD in Saudis. Permission from principal investigator was taken to work on this academic research project. Multi-stage random sampling was conducted in 13 regions of Kingdom of Saudi Arabia (KSA). Urban and rural areas of each region were considered as strata. Thus, out of 1623 primary health care centers (PHCS) in KSA, 58 were randomly selected from urban strata and 66 PHCS were selected from rural strata. Second stage cluster sampling was performed while selecting household from urban and rural community. A ratio of 2:1 was used for selecting the same. Intuitively, 100 households from urban and 50 households from rural community were selected as sampling units. A total of 17232 subjects were sampled by multi-stage random sampling of households and PHCS distributed in urban and rural areas of 13 regions in KSA.

Subjects of both genders between 30-70 years of ages were included in this study. A detailed questionnaire was also filled which included questions related to history and current activities of the participants. They were interviewed about their demographic profile and investigation of risk factors associated with CAD was also made. Demographic profile comprised of questions about age, gender, marital status, education status, monthly household income, occupation and housing type. Family history, exercise frequency, smoking status, presence of any other symptoms and number of times of hospital admission were the questions asked for assessing probable risk factors of CAD. Height, weight and other anthropometric measurements were taken for physical examination. Moreover, clinical diagnoses were performed by well-trained physicians. These diagnoses included lipid tests, electrocardiography (ECG), exercise tests, coronary angiography, blood pressures, glucose levels etc.

The patients were asked about when they had their first chest pain in past. The number of patients who were diagnosed to have CAD was established in finding of one or more of the following criteria: either physician’s clinical assessment of the chest pain as angina, previous myocardial infarction (MI), or findings of evidence of previous MI by ECG. An article published in this series reported the prevalence of CAD in KSA was approximately 6%.^13^ Age, gender, body mass index (BMI)^14^, waist circumference (WC)^15^, systolic &amp; diastolic blood pressures (SBP &amp; DBP)^16^, fasting blood sugar (FBS)^17^, serum cholesterol (S. Chol.), serum triglycerides (S.TG) and high density lipoprotein (HDL)^18^ were the prominent associated factors with CAD among Saudi population. This article is the series of earlier publication of the same research on assessing prevalence of CAD and its risk factors.

### Statistical analysis

The statistical analyses were conducted using Stata v. 12.0. The data were converted from snapshot (cross sectional) to time dependent. Duration of chest pain was set as time variable. CAD presence in the patients was set as event variable. The patient who had chest pain in past but had not diagnosed with CAD were censored. The patients, who were diagnosed as CAD patients, had not chest pain in past were truncated. Descriptive duration analysis was computed in terms of mean ± standard deviation and median (inter-quartile range). Frequency and percentages were computed for categorical variable. Bivariate association of factors with CAD among patients with chest pain was assessed by Chi-square test. Kaplan-Meier duration analysis was executed to estimate duration of chest pain on different time span. The duration of chest pain was compared in study population who were adhered with different status of significant risk factors. Mann-Whitney U test and Kruskal-Wallis test followed by Tukey’s type non-parametric post-hoc test were employed for the factors with two and at-least three categories respectively. Stratification with the status of CAD presence was also done and two-way non-parametric analysis of variance was run to study effect of risk factors on duration of CAD. To predict the duration of chest pain to CAD in presence of factors and covariates with p value less than 0.20 at univariable analyses, stepwise Cox regression model was run while setting p values of stay and removal as 0.05 and 0.10 respectively. The accuracy of model was checked by Receiver Operating Characteristic (ROC) analysis. Somers’ D coefficient was calculated to assess magnitude of accuracy of the model.

## Results

### Descriptive duration analysis

**Figure 1:**
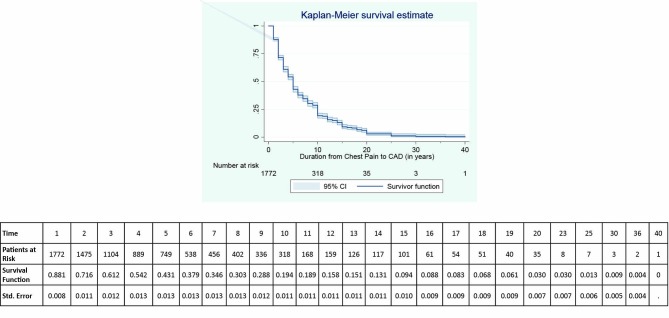
Kaplan-Meier Curve portraying duration from chest pain to CAD in terms of Survivor function with 95% confidence interval.

Out of 17232 patients, 24.1% (n = 4157) endured chest pain in past. Cardiac chest pain was experienced by 884 (85.3%) patients out of 1,036 CAD patients. This also reckoned that 21.2% (884/4157) of patients with chest pain experienced coronary artery disease. The shortest duration from chest pain to the diagnosis of CAD was 1 year and the longest was 40 years. The median duration was 5 years with 2 and 10 were 1^st^ and 3^rd^ quartiles of the same respectively. Kaplan-Meier estimator revealed that 88% of the patients with chest pain did not experience CAD by 1^st^ year (95% CI: 86.5%–89.5%). This endurance reached about half of the above proportion (43%) by 5 years of the duration. All the patients with 17 years of chest pain were diagnosed as CAD patients with duration function of 8.3% (95% CI: 6.6%–10.2%). A similar panorama is suffered by the CAD patients with duration of at least 25 years of chest pain with very low duration function (Figure 1).

### Stratified duration analysis

**Figure 2:**
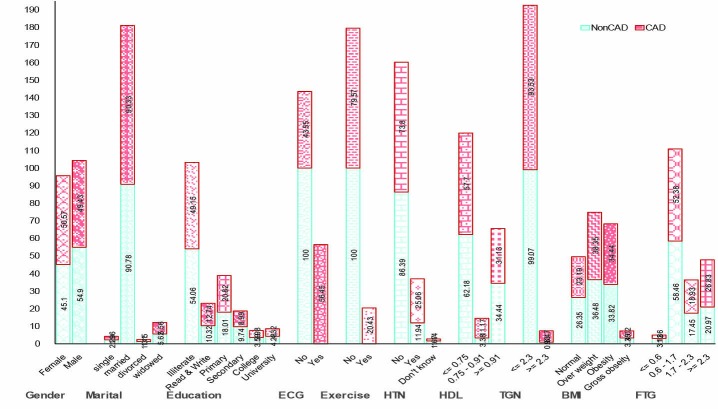
Presence of CAD risk factors in patients with chest pain in past.

About 24% of the patients with chest pain endured CAD (Figure 2). The proportion of CAD was observed significantly more in female (P=0.004), those who did not exercise (P<0.0001), hypertensive (P<0.0001), patients having low HDL (P<0.0001), less triglyceride nitrate (P<0.0001) and low fasting triglyceride (P<0.0001).

Table 1 indicated effect of these demographic and clinical risk factors on duration from chest pain to CAD. The mean duration of chest pain for the patients who exercised was 0.55 ± 2.23 which is less than those who did not exercise (P<0.0001). The male patients with chest pain diagnosed with CAD 5 months earlier than female patients (P=0.003). Similarly, diagnosis of CAD for the patients with less than 2.3 triglycerides nitrates delayed more than those who had more than 2.3 values of the same (P<0.005).

**Table 1:** Distribution of duration in different demographic and clinical characteristics*

**Factors**	**Categories**	**Mean**	**SD**	**Median**	**IQR**	**Min.**	**Max.**	**P-value**
**Exercise**	No	1.8	3.7	0	2	0	40	<0.0001
	Yes	0.55	2.23	0	0	0	20	
**Hypertension**	No	1.8	3.7	0	2	0	40	0.514
	Yes	1.5	3.5	0	1	0	25	
	Not certain	0.5	1.5	0	0	0	7	
**Gender**	Female	1.95	3.95	0	2	0	40	0.003
	Male	1.6	3.35	0	2	0	36	
**HDL**	≤0.75	1.9	3.8	0	2	0	40	0.133
	0.75 - 0.91	0.97	2.9	0	0	0	23	
	≥0.91	1.6	3.4	0	2	0	20	
**TGN**	≤2.3	1.8	3.6	0	2	0	40	<0.005
	≥2.3	1.1	3.7	0	0	0	23	
BMI**	Normal	1.75	3.7	0	2	0	40	0.394
		Overweight	1.8	3.7	0	2	0	36	
	Obese	1.8	3.55	0	2	0	30	
	Gross obese	1.3	3.2	0	1	0	20	
**FTG**	≤0.6	1.7	3.7	0	1	0	20	0.507
	0.6 - 1.7	1.7	3.6	0	2	0	40	
	1.7 - 2.3	2.0	3.9	0	2	0	25	
	≥2.3	1.7	3.6	0	2	0	30	

*IQR: Interquartile range, Max.: Maximum value, Min.: Minimum value, SD: Standard deviation, **BMI: Normal (BMI < 25 KG/m^2^), Overweight (BMI 25–30 KG/m^2^), Obese (BMI 30–40 KG/m^2^), Gross obese (BMI >40 KG/m^2^)

Comparing the duration from chest pain in those participants who either suffered CAD or not, the patients with CAD had shorter duration (Table 2). Hypertensive patients had longer duration of encountering CAD than normotensive (P=0.002) whereas patients without CAD had less duration of being hypertensive and suffering chest pain (P=0.002). Male has less duration of chest pain in either cases (CAD or without CAD) (P=0.013). Patients with lowest and highest HDL diagnosed with CAD within half of the year once encountering chest pain while normal HDL delayed the same for about 9 months. This panorama for patients without CAD observed for about 2 years and 1 year respectively (P=0.034). When triglyceride was less than 2.3 the duration of chest pain for CAD free patients was 2 years and for CAD patients, it was 0.54 years (Table 2). The increased BMI shortened the duration from chest pain to CAD for both type of patients (P<0.001). On the other hand, increased FTG prolonged the event of CAD as patients having FTG ≤0.6 diagnosed with CAD prior to 2 years (P<0.0001).

**Table 2:** Distribution of duration in different demographic and clinical characteristics stratified with presence of CAD*

**Factors**	**Categories**	**No**						**Yes**						**P Value**	
		**Mean**	**SD**	**Median**	**IQR**	**Min.**	**Max.**	**Mean**	**SD**	**Median**	**IQR**	**Min.**	**Max.**	**Factors**	**CAD**
**Exercise**	No	2.1	3.9	0	3	0	40	0.6	2.3	0	0	0	23	0.473	-
	Yes							0.55	2.2	0	0	0	20		
**Hypertension**	No	2.2	3.9	0	3	0	40	0.5	2.0	0	0	0	20	0.659	0.002
	Yes	1.9	3.8	0	2	0	25	0.8	2.9	0	0	0	23		
	Not certain	0.6	1.6	0	0	0	7								
**Gender**	Female	2.35	4.2	0	3	0	40	0.8	2.8	0	0	0	23	0.072	0.013
	Male	1.9	3.6	0	2	0	36	0.4	1.6	0	0	0	15		
**HDL**	≤0.75	2.3	4.05	0	3	0	40	0.6	2.05	0	0	0	20	0.435	0.034
	0.75 - 0.91	1.0	2.4	0	1	0	10	0.9	3.3	0	0	0	23		
	≥0.91	1.9	3.6	0	2	0	20	0.5	2.2	0	0	0	20		
**TGN**	≤2.3	2.1	3.9	0	3	0	40	0.5	2.1	0	0	0	20	0.652	0.265
	≥2.3	1.1	2.75	0	0	0	10	1.1	4.0	0	0	0	23		
**BMI**	Normal	2.0	3.9	0	3	0	40	2.2	4.0	0	3	0	36	0.503	<0.0001
	Overweight	2.2	4.0	0	3	0	36	2.1	3.7	0	3	0	30		
	Obese	2.1	3.7	0	3	0	30	1.6	3.4	0	1	0	20		
	Gross Obese	1.6	3.4	0	1	0	20	0.6	2.4	0	0	0	20		
**FTG**	≤ 0.6	1.95	3.9	0	2	0	20	1.99	3.8	0	3	0	40	0.090	<0.0001
	0.6 - 1.7	1.99	3.8	0	3	0	40	2.4	4.2	0	3	0	25		
	1.7 - 2.3	2.4	4.2	0	3	0	25	2.12	3.95	0	3	0	30		
	≥2.3	2.2	3.95	0	3	0	30	0.6	2.35	0	0	0	20		

*IQR: Interquartile range, Max.: Maximum value, Min.: Minimum value, SD: Standard deviation

### Duration Model

Crude and Adjusted hazard ratio obtained by duration model are shown in Table 3 and Table 4 respectively. Table 3 indicated that age was not significantly hazardous for the patient with chest pain. Female had 14% less hazard to endured CAD than males (P=0.004). Hypertension and diabetes had also not significant hazard for patient of chest for having CAD. Others risk factors which were significant on overall status of chest pain patients were not found to be significant in univariable analysis for the hazards of bearing CAD. Multivariable Cox regression via stepwise method revealed different prospect for the hazards of CAD for the patient with chest pain. It stipulated that when effect of other factors accounted for, female had 9% times less likely to have CAD, increased age and exercise delayed the same hazard and higher values of HDL reduced the hazard for chest pain’s patients for encountering CAD.

**Table 3:** Univariable Cox regression analysis estimating duration by different characteristics of patients

**Factor**	**Categories**	**Hazard ratio**	**S.E. (HR)**	**Z**	**P > |z|**	**95% CI**		**Log-Likelihood**	**Likelihood Ratio Test**	**P > Chi-square**
**Age, years**	** **	1.0	0.002	-1.32	0.19	0.99	1.002	-8471.6	1.75	0.186
**Gender**	Female	0.85	0.05	-2.86	0.004	0.76	0.95	-8605.3	8.20	0.004
	Male	1								
**Hypertension**	No	0.91	0.24	-0.38	0.71	0.55	1.51	-8531.1	4.42	0.110
	Yes	0.75	0.21	-1.04	0.29	0.44	1.29			
	Not Certain	1								
**Diabetes**	No	1.29	0.28	1.20	0.23	0.85	1.98	-8523.4	3.33	0.189
	Yes	1.17	0.26	0.68	0.49	0.75	1.81			
	Not Certain	1								
**HDL**	No	1.13	0.07	1.89	0.06	0.99	1.27	-8549.4	10.30	0.001
	Yes	0.69	0.13	-1.89	0.06	0.48	1.01			
	Not Certain	1								
**TGN**	≤2.3	2.14	0.76	2.14	0.03	1.07	4.27	-8237.5	5.98	0.014
	≥ 2.3	1								
**BMI**	Normal	0.99	0.17	-0.04	0.97	0.71	1.39	-8553.5	0.02	0.999
	Overweight	0.99	0.17	-0.08	0.94	0.71	1.37			
	Obese	0.985	0.17	-0.09	0.93	0.71	1.37			
	Gross obese	1								
**FTG**	≤0.6	1.03	0.18	0.15	0.88	0.73	1.45	-8365.6	0.41	0.939
	0.6 - 1.7	1.045	0.07	0.62	0.54	0.91	1.20			
	1.7 - 2.3	1.04	0.09	0.48	0.63	0.88	1.24			
	≥ 2.3	1								

**Table 4:** Predictors for the Hazards during Duration from Chest Pain to CAD

**Factors**	**Hazard ratio**	**SE (HR)**	**Z**	**P < |Z|**	**95% CI**	**Log-Likelihood**	**Likelihood Ratio Test**	**P > Chi-Sq**
Gender (Female/Male)	0.91	0.04	-2.55	0.01	0.85	0.98	-23283.4	22.01	0.0002
Age, years	1.003	0.002	1.79	0.07	1.0	1.01			
Exercise (No/Yes)	1.31	0.09	3.03	0.002	1.10	1.57			
HDL(No/Yes)	0.92	0.04	-2.24	0.025	0.855	0.99			

**Figure 3:**
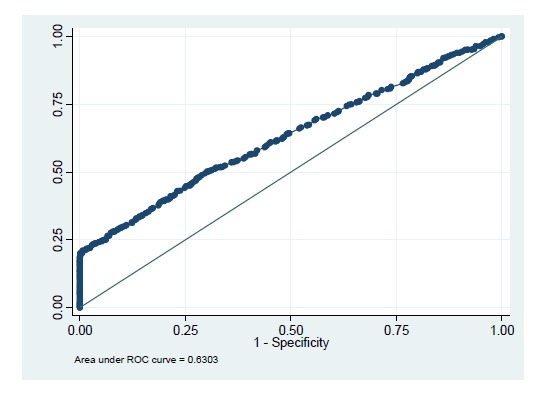
Receiver operating characteristics curve showing accuracy of prediction model

The area under the receiver operating characteristics curve was 64% (Figure 3) indicating moderate accuracy of stepwise model. Somer’s D coefficient was also depicted significant magnitude of accuracy of the model (D= 0.261; 95% CI: 0.211–0.310).

## Discussion

The population of KSA includes inhabitants from various countries.^19,20^ These individuals endure noteworthy prevalence of CAD.^13^ Several studies have been done for studying risk factors of CAD.^21-24^ Wilson et al developed predicted score for the risk of coronary heart disease^25^ which is considered to be generalized score for any cardiac patients.^11^ The score also predicted first coronary events^26^ and determined highest risks of coronary events in elderly age of both men and women. Later, prediction scores for coronary disease via logistic model were built for primary care patient with acute chest pain.^9,10,12^ Duration of symptom was considered to be the significant predictors in the model.^12^ Furthermore, duration of mortality due to CAD was predicted in Australian population.^27,28^ Mingala and Estolano made an attempt to estimate duration of hospital stay for patients suffering MI.^29^ As far as authors’ knowledge is concerned, the time duration from the first onset of chronic chest pain to the true diagnosis of CAD has never been studied yet.

For the analysis of duration data, two study designs have been recommended cross sectional or cohort study designs. Though, cohort study requires time to observe complete duration while cross sectional study needs knowledge of duration of disease for the currently enrolled patients.^30^ Current duration survival analysis was introduced by Keiding et al ^31^ and Ali et al ^32^. These gaps incited the idea that we exerted an attempt to analyze the duration of CAD from chest pain from a cross sectional sample.

The study estimated average duration from chest pain to the diagnosis of CAD as 5 years. Mingala and Estolano reported the average duration of hospitalization for CAD patients was 0.5 years.^29^ The average duration of CAD mortality was varied from 10 years^33^ to 25 years.^34^ Combining these outcomes, we can say now that duration from chronic chest pain to the CAD mortality varied from 15.5 years to 30.5 years.

Hermingway et al studied prevalence of angina across 31 countries by systematic review. The study reported average weighted prevalence in female was 6.7% while in male, it was 5.7%. In population of Iran, the same prevalence was found to be 4.3%.^3^ The prevalence of chest pain in Saudi adults is found to be 24% by this study. This prevalence is also relatively high in male and female residing in KSA.

Sarraf-Zadegan et al reported that in Iran cardiac chest pain leading to CAD endured by 19.4% of population.^4^ By our study, it was found that 85.3% Saudi population encountered cardiac chest pain leading to CAD. Though, in primary care^6^, study identified 8.2% of cases as CAD in a cohort with chest pain. A study from United States indicated 83.3% prevalence of CAD among patients with chest pain.^35^ Similarly, a 300 weeks follow up study^5^ reported that CAD was prevalent in 12% of the patients with chest pain. This study also advised that great considerations should be made on patients with chest pain as in this study out of 24620 cohort of patients with chest pain, 672 (2.7%) diagnosed with any one of the thoracic ailments.

Amongst the known risk factors for the development of coronary artery disease, this study indicated certain predictors which were hazardous for the chest pain patients concerning the same disease. Increasing age is significantly hazardous during the time span from chest pain to CAD. Though, this effect is slight similar as reported for overall population at risk of CAD in the earlier article.^13^ Advancing age and gender had also considered as significant risk factors for CAD among Iranian population.^36^ Exercise was not a significant predictor by regression model in the same study. However, in our study increasing exercise showed significant hazards for CAD for the patients who encountered chest pain. This indicated that patient who encountered chest pain should be vigilant for their exercise habits. These findings can be comparable with another study by Bosner et al. in which age/gender with exercise were the leading predictors for the development of CAD among primary care patients with acute chest pain.^10^ HDL was the only metabolic syndromes affected the hazards of CAD to the chest pain patients. The accuracy of the model is moderately good and magnitude of accuracy is acceptable. This indicated that stepwise Cox model for studying duration of chest pain is good enough to apply.

## Conclusion

The duration from chest pain to CAD was computed to be 5 years. Age, gender, exercise, and HDL were the significant predictors that best describe the distribution of duration from chest pain to CAD. Physicians should prioritize these factors as variable of concern for the patient with chest pain to avoid risk of having CAD in long haul.

## Ethical issues

Approval of study was obtained from Board of Advanced Study and Research of the institute.

## Competing interests

Authors declare no conflict of interests in this study.
